# Genetic diversity of the O antigens of *Proteus* species and the development of a suspension array for molecular serotyping

**DOI:** 10.1371/journal.pone.0183267

**Published:** 2017-08-17

**Authors:** Xiang Yu, Agnieszka Torzewska, Xinjie Zhang, Zhiqiu Yin, Dominika Drzewiecka, Hengchun Cao, Bin Liu, Yuriy A. Knirel, Antoni Rozalski, Lei Wang

**Affiliations:** 1 Key Laboratory of Molecular Microbiology and Technology of the Ministry of Education, TEDA College, Nankai University, Tianjin, P. R. China; 2 TEDA Institute of Biological Sciences and Biotechnology, Nankai University, Tianjin, P. R. China; 3 Tianjin Research Center for Functional Genomics and Biochips, TEDA College, Nankai University, Tianjin, P. R. China; 4 Tianjin Key Laboratory of Microbial Functional Genomics, TEDA College, Nankai University, Tianjin, P. R. China; 5 Department of Immunobiology of Bacteria, Department of General Microbiology Institute of Microbiology, Biotechnology and Immunology, Faculty of Biology and Environmental Protection, University of Lodz, Lodz, Poland; 6 N.D. Zelinsky Institute of Organic Chemistry, Russian Academy of Sciences, Moscow, Russian Federation; University of Helsinki, FINLAND

## Abstract

*Proteus* species are well-known opportunistic pathogens frequently associated with skin wound and urinary tract infections in humans and animals. O antigen diversity is important for bacteria to adapt to different hosts and environments, and has been used to identify serotypes of *Proteus* isolates. At present, 80 *Proteus* O-serotypes have been reported. Although the O antigen structures of most Proteus serotypes have been identified, the genetic features of these O antigens have not been well characterized. The O antigen gene clusters of *Proteus* species are located between the *cpxA* and *secB* genes. In this study, we identified 55 O antigen gene clusters of different *Proteus* serotypes. All clusters contain both the *wzx* and *wzy* genes and exhibit a high degree of heterogeneity. Potential functions of O antigen-related genes were proposed based on their similarity to genes in available databases. The O antigen gene clusters and structures were compared, and a number of glycosyltransferases were assigned to glycosidic linkages. In addition, an O serotype-specific suspension array was developed for detecting 31 *Proteus* serotypes frequently isolated from clinical specimens. To our knowledge, this is the first comprehensive report to describe the genetic features of *Proteus* O antigens and to develop a molecular technique to identify different *Proteus* serotypes.

## Introduction

*Proteus* species are gram-negative bacterial opportunistic pathogens that belong to the Enterobacteriaceae family [1]. The genus *Proteus* is widely distributed in the natural environment and in the microflora of human and animal intestines [1]. Under favorable conditions, they most commonly cause skin wound and urinary tract infections (UTIs) in humans and animals [2–4], and roles in rheumatoid arthritis have also been reported [5]. Currently, this genus contains seven named species, *P*. *mirabilis*, *P*. *penneri*, *P*. *vulgaris*, *P*. *myxofaciens*, *P*. *hauseri*, *P*. *terrae*, and *P*. *cibarius* as well as three unnamed *Proteus* genomospecies, 4, 5, and 6 [6–9]. Among these, *P*. *mirabilis*, *P*. *penneri*, and *P*. *vulgaris* are the most common human pathogens, and isolates of *P*. *mirabilis* cause UTIs with the highest frequency [4]. *P*. *myxofaciens*, *P*. *terrae*, and *P*. *cibarius* have no pathogenicity for humans [3, 8, 10].

*Proteus* species express a series of virulence factors that are associated with infection processes and disease, such as fimbria, flagella, hemolysins, urease, proteases, amino acid deaminases, lipopolysaccharide (LPS) and capsular polysaccharides (CPSs) [3, 11–13]. LPS is an endotoxin that constitutes the outer cell membrane of the gram-negative bacteria and is its most variable component [14]. LPS is thought to play an important role in the process of the UTIs and to affect both bladder and kidney stone formation [15–17]. Furthermore, LPS confers protection against serum-mediated bactericidal activity to bacteria [18].

LPS consists of three parts: a lipid A anchor, a core oligosaccharide, and an O-specific polysaccharide (OPS) [19]. The OPS consists of oligosaccharide repeating units (O units) that usually contains 2 to 8 sugar residues [20]. The OPS is the most variable component of the LPS, which defines the serological specificity of gram-negative bacteria [19, 21]. OPS variation is predominantly determined by the types of sugars present as well as the order of sugar residues and the linkages between them [21, 22]. The OPS is essential for bacterial survival, and the loss of OPS causes many bacteria serum-sensitive or affects their virulence in another way [21, 23].

The OPS synthesis related genes usually form a gene cluster that is located at a fixed position on the chromosome [19, 21]. For example, in *Salmonella*, *Escherichia coli*, and *Shigella*, the O antigen gene clusters most commonly map between the *galF* and *gnd* genes [19, 21]. One or more of the genes involved in OPS synthesis may sometimes map outside the gene clusters [19, 21]. The O antigen gene clusters contain three classes of genes: nucleotide sugar biosynthesis pathway genes, glycosyltransferase (GT) genes, and O antigen processing genes [19, 21, 23]. Sugars commonly found in other polysaccharide structures or involved in metabolism, such as galactose (Gal), glucose (Glc), and *N*-acetylglucosamine (GlcNAc), are usually synthesized by enzymes encoded by genes outside the O antigen gene cluster [19, 21]. There are three different pathways to synthesize and translocate O antigen: the Wzx/Wzy pathway, which is most frequently utilized; the synthase pathway; and the ATP-binding cassette transporter pathway (ABC pathway) [19, 21, 24]. In the Wzx/Wzy pathway, the O units are synthesized by initial transfer of a sugar phosphate, then sequential transfer of the other sugars from their nucleotide sugars donor to the carrier undecaprenyl phosphate (UndP) [25]. The assembled O units are flipped across the cell membrane by Wzx and then polymerized by Wzy to form polysaccharide chains [25]. In *E*. *coli*, *Shigella*, and *Salmonella*, the chain-length determinant Wzz imposes a modal chain-length distribution on the OPS, loss of *wzz* results in the uncontrolled polymerization of O-units by Wzy-producing nonmodal chain-lengths ranging from short to long, the principle is still unknown [26]. The OPS is eventually ligated to the lipid A core to form LPS [27].

Detection of bacterial serotypes is critical for prevention and control of pathogens. However, traditional antiserum serotyping methods are laborious and cross-reactive, many molecular and chemical serotyping techniques have been developed. Such as real-time PCR assays and short sequencing assays based on serotype-specific genes [28–29], or chemometric analysis of attenuated total reflectance infrared spectra based on defined LPS structures [30]. Recent development of gene chip technology, including solid phase arrays and liquid bead-based suspension arrays, has given us a more sensitive and accurate method to identify bacterial serotypes [31]. The suspension array system is based on microspheres labeled with a unique dye combination. The microspheres are coupled with specific probes for targets which are amplified from the samples using biotin-labeled primers. The fluorescent emission of the target analyte is measured by exciting the fluorescent reporter bound to the microspheres [31].

Currently, according to OPS diversity, *Proteus* species are divided into 80 different serotypes [32–34]. We previously reported 5 O antigen gene clusters (O3ab, O10, O23ac, O27, and O47) [35]. In this study, we identified 55 new O antigen gene clusters. Collectively, we have thus characterized 60 O antigen gene clusters from different *Proteus* serotypes (O1-O3, O5-O6, O8-O14, O16-O21, O23-O34, O36-O37, O40-O42, O44-O45, O47-O48, O50-O62, O65, O67, O69, and O71-O75). All these clusters are located between *cpxA* and *secB* and contain both *wzx* and *wzy* genes. They include genes associated with nucleotide sugar biosynthesis, sugar transfer, O antigen processing, and several other genes. In most cases, the O antigen gene clusters correspond to known O antigen structures. The *wzx* and *wzy* genes in these 60 *Proteus* strains were polytropic, which provided a basis for rapid molecular serotyping. Using the *wzx* and *wzy* genes, we developed a PCR-based suspension array to distinguish 31 different *Proteus* serotypes (O1-O3, O5-O6, O8-O14, O17-O21, O23-O24, O27, O29-O34, O36, O40, O42, O45, and O47), which are frequently isolated from clinical specimens. More serotypes can be added to this array in the future by designing new probes based on their *wzx* or *wzy* genes.

## Materials and methods

### Bacterial strains

All of the *Proteus* strains used in this study are shown in S1 Table, which were provided by the Department of Immunobiology of Bacteria and the Department of General Microbiology at the Institute of Microbiology, Biotechnology and Immunology, Faculty of Biology and Environmental Protection, University of Lodz (Lodz, Poland).

### Genomic DNA extraction and O antigen gene cluster amplification

The *Proteus* strains were grown in Luria Broth and then harvested by centrifugation [36]. Genomic DNA samples were isolated using a Bacteria Extraction Kit (CWBIO Co., Ltd, China). Primers wl_31262 (5’-GAGTTATTACGHGAAACGGTAAAAGC-3’) and wl_31263 (5’-GTTAACTTTGATGCGTTGTTTATGAACTA-3’) designed based on the *cpxA* and *secB* genes, respectively, were used to amplify the *Proteus* O antigen gene clusters [35]. The PCR program used was as follows: an initial denaturation at 95°C for 3 min, followed by 30 cycles of denaturation at 95°C for 45 s, annealing at 55°C for 45 s, and extension at 68°C for 15 min with a final extension at 68°C for 5 min [35].

### O antigen gene cluster sequencing and analysis

The PCR products were fragmented with DNase I, then the fragments were cloned into pGEM-T Easy vector to construct a library as described previously [37]. Sequencing was performed using an ABI 3730 automated DNA sequencer (Applied Biosystems, Foster City, CA), with 12–20 fold coverage of the O antigen gene clusters. Sequencing data were assembled using the Staden package and were annotated by Artemis [38, 39]. Use TBLAST and PSI-BLAST to search available database, including the Pfam protein database and the GenBank database, and to identify potential functions of the O antigen synthesis related genes [40]. The potential transmembrane segments were identified using the TMHMM 2.0 program [40]. The GT genes were divided into homology groups (HGs) using the OrthoMCL program v2.0 [41] with a 50% protein sequence identity used as the cut-off. ClustalW v2.0 was used for sequences alignment, and JC69 module and phyML v3.0 were used to construct maximum likelihood trees [42].

### Development of PCR system

DNA from different samples was amplified using the Hot Start PCR Kit (Promega, Madison, WI). PCR primers designed based on specific *wzx/wzy* genes were used to generate PCR fragments of 100 to 495 bp (S2 Table) [43]. The reverse primer was biotinylated at the 5’-end that can be combined with microspheres coupling the dye streptavidin-R-phycoerythrin. The median fluorescence intensities (MFI) were detected using the Bio-Plex 100 suspension array system (Bio-Rad). A single multiplex PCR system was used to amplify the *wzx/wzy* genes as follows: an initial denaturation at 95°C for 5 min, followed by 30 cycles of denaturation at 94°C for 45 s, annealing at 50°C for 1 min, and extension at 72°C for 1min, and the final extension was done at 72°C for 10 min [31]. The PCR products were then used directly in the hybridization reaction to couple beads.

### Probes design and beads coupling

Serotype-specific probes were designed based on the *wzx/wzy* genes (S3 Table). BioEdit software 7.0 version was used for multiple sequence alignments. The carboxylated beads (Bio-Rad, Hercules, CA) were coupled to specific probes with an amino C-12 modifiication at the 3’-end (AuGCT, China).

### Hybridization and staining

17 μl of the biotinylated amplicon was mixed with 33 μl of the bead mixture containing 2,500 beads in a 1.5× tetramethylammonium chloride (TMAC) solution (Sigma, St. Louis, MO). Then the mixture was denatured at 95°C for 5 min followed by hybridization at 55°C for 15 min. The hybridization product was collected using centrifugation at 8000 rpm and resuspended using 75 μl 1× TMAC solution containing 10 ng/mL streptavidin-R-phycoerythrin (Molecular Probes, Eugene, OR), then incubated at 55°C for 10 min.

### Data acquisition and analysis

The fluorescence intensities of the beads were analyzed using a Bio-Plex 100 suspension array system (Bio-Rad). The MFIs were calculated from 100 replicate measurements using the digital signal processor and the Bio-Plex Manager software 4.1. A positive result was defined as an MFI > 150 and a signal/background (S/B = MFI/Blank) > 6.0 [31].

### Results and discussion

**Fig 1:**
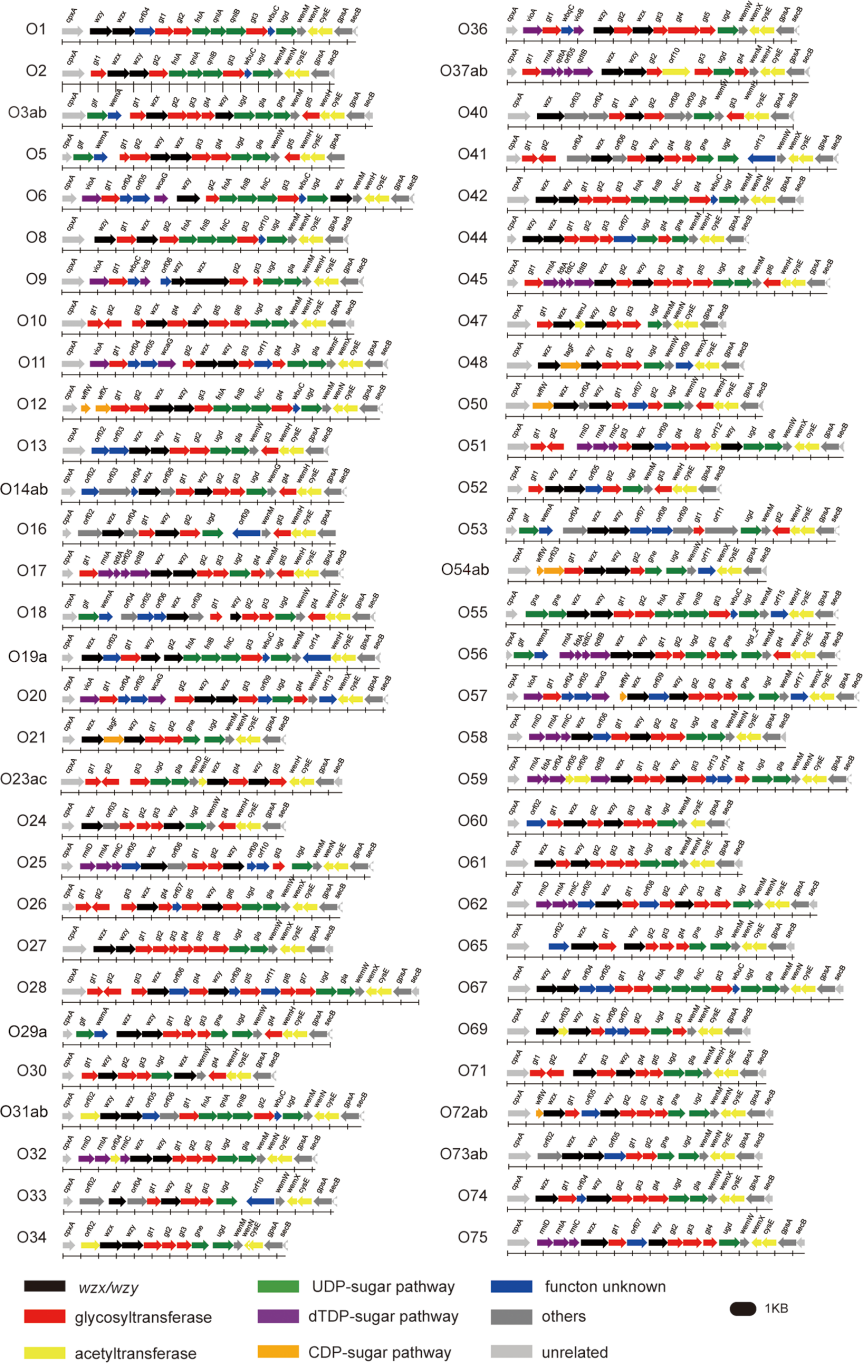
The O antigen gene clusters from the 60 *Proteus* serotypes. The sequences of the 60 *Proteus* O antigen gene clusters have been deposited to GenBank with accession numbers KY710685 to KY710739.

In this study, we identified 55 new *Proteus* O antigen gene clusters. Combined with the 5 previously published gene clusters (O3ab, O10, O23ac, O27, and O47) [35], we have a total of 60 *Proteus* O antigen gene clusters characterized. All these clusters are located between *cpxA* and *secB* and contain both *wzx* and *wzy* genes, various GT genes, and nucleotide sugar synthesis genes (Fig 1). The GC content of these O antigen synthesis related genes ranged from 19.5 to 35.7%, which is lower than the rest of the *Proteus* genome (38.9%) [44], indicating that the O antigen gene clusters of *Proteus* species may originate from other bacteria [35].

### Comparison of *Proteus* O antigen gene clusters and structures

**Fig 2:**
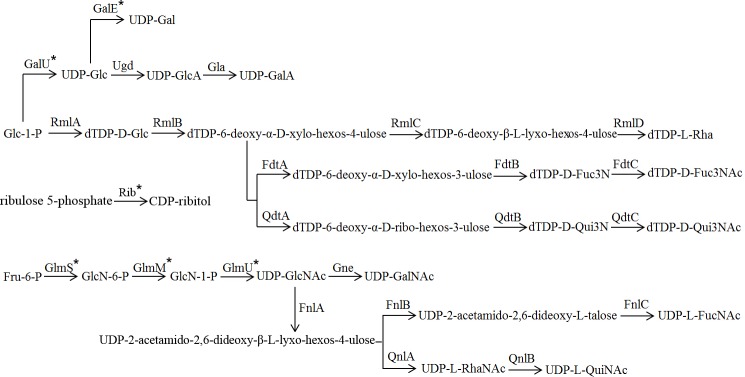
Biosynthesis pathways for the sugars in *Proteus* O antigens. GalU, UTP-glucose-1-phosphate uridylyltransferase; GalE, UDP-glucose-4-epimerase; Ugd, UDP-glucose 6-dehydrogenase; Gla, UDP-glucuronate 4-epimerase; GlmS, glutamine:fructose-6-phosphate transaminase; GlmM, phosphoglucosamine mutase; GlmU, UDP-*N*-acetyl-glucosamine pyrophosphorylase; Gne, UDP-*N*-acetylglucosamine-4-epimerase; RmlA, glucose-1-phosphate thymidylyltransferase; RmlB, dTDP-D-glucose 4,6-dehydratase; RmlC, dTDP-6-deoxy-α-D-*xylo*-hexos-4-ulose 3,5-epimerase; RmlD, dTDP-6-deoxy-β-L-*lyxo*-hexos-4-ulose 4-reductase; FdtA, dTDP-6-deoxy-α-D-*xylo*-hexos-4-ulose 3,4-isomerase; FdtB, dTDP-6-deoxy-α-D-*xylo*-hexos-3-ulose aminase; FdtC, dTDP-D-Fuc3N acetylase; QdtA, dTDP-6-deoxy-α-D-*xylo*-hexos-4-ulose 3,4-isomerase; QdtB, dTDP-6-deoxy-α-D-*ribo*-hexos-3-ulose aminase; QdtC, dTDP-D-Qui3N acetylase; FnlA, UDP-D-GlcNAc 4,6-dehydratase, 3- and 5-epimerase; FnlB, UDP-2-acetamido-2,6-dideoxy-β-L-*lyxo*-hexos-4-ulose 4-reductase; FnlC, UDP-2-acetamido-2,6-dideoxy-L-talose 2-epimerase; QnlA, UDP-2-acetamido-2,6-dideoxy-β-L-*lyxo*-hexos-4-ulose 4-reductase; QnlB, UDP-L-RhaNAc 2-epimerase; * indicates the genes located outside the O antigen gene cluster (all enzymes encoded by these genes can be found in the 68 *Proteus* genomes; the amino acid sequence identities of GalU to the homolog in *E*. *coli* K12 are 75.44–76.43%, the amino acid sequence identities of GalE to the homolog in *E*. *coli* K12 are 59.13–65.26%, the amino acid sequence identities of Rib to the homolog in *E*. *coli* K12 are 94.29–94.6%, the amino acid sequence identities of GlmS to the homolog in *E*. *coli* K12 are 78.89–82.79%, the amino acid sequence identities of GlmM to the homolog in *E*. *coli* K12 are 80.97–88.12%, the amino acid sequence identities of GlmU to the homolog in *E*. *coli* K12 are 73.94–79.8%, data not shown).

Genes related to the biosynthesis of common sugar nucleotide precursors (such as UDP-GlcNAc, UDP-Glc, and UDP-Gal) are not located in the O antigen gene cluster [20, 22]. Genes related to the biosynthesis of rare monosaccharide precursors (such as UDP-QuiN, UDP-FucN, dTDP-Qui3N, dTDP-Fuc3N, dTDP-Rha) were typically located in the O antigen gene cluster [19, 21]. Based on these features, we compared the O antigen gene clusters and structures in *Proteus* (Figs 1 and 2, S4 Table) [32]. Of the 60 *Proteus* O antigen gene clusters, 57 (95% of the collection) were found to correspond to their known O antigen structures. Next, the genetic and structural consistency of rare monosaccharides will be described.

There are 4 *Proteus* O antigens whose structures include QuiNAc (O1, O2, O31, and O55), and their gene clusters all contain *fnlA-qnlAB*. There are 6 *Proteus* O antigens whose structures include FucNAc (O6, O8, O12, O19a, O42, and O67), and their gene clusters all contain *fnlABC*. UDP-GlcNAc is converted to UDP-2-acetamido-2,6-dideoxy-β-L-*lyxo*-hexos-4-ulose by *fnlA* [45], which can be further converted to UDP-L-QuiNAc by *qnlAB* or to UDP-L-FucNAc by *fnlBC* [45–47].

There are 2 *Proteus* O antigens whose structures include Fuc3NAc (O17 and O45). The O45 gene cluster contains *rmlA-fdtABC*, and the O17 gene cluster contains *rmlA-fdtAB*. There are 2 *Proteus* O antigens whose structures include Qui3NAc (O56 and O59), and their gene clusters both contain *rmlA-qdtABC*. There are 7 *Proteus* O antigens whose structures include Rha (O25, O32, O51, O55, O58, O62, and O75), and all corresponding gene clusters except that of O55 contain *rmlACD*. Glc-1-P is converted to dTDP-6-deoxy-α-D-*xylo*-hexos-4-ulose by *rmlAB* [48, 49], which is further converted to dTDP-L-Rha by *rmlCD* [50, 51] or dTDP-D-Fuc3NAc by *fdtABC* [52] or dTDP-D-Qui3NAc by *qdtABC* [53]. However, we did not find the *rmlB* gene in any of these *Proteus* O antigen gene clusters, indicating that the *rmlB* gene may be located outside the clusters in these strains. Therefore, we downloaded 68 *Proteus* genomes (S5 Table), and identified the RmlB in all these genomes, which had amino acid sequence identities to the homolog in *E*. *coli* K12 from 71.95% to 74.76% (data not shown).

There are 4 *Proteus* O antigens whose structures include Rib*f* (O9, O25, O36, and O59), the only pentose, which is available from the NAD salvage pathway [54]. The gene responsible for the synthesis of UDP- Rib*f* is not always located in O antigen gene clusters [19, 21], and we did not find it in these strains too. There are 5 *Proteus* O antigens whose structures include Rib-ol (O16, O33, O41, O53, and O73ab), which is synthesized from ribulose 5-phosphate by *rib* [54]. However, we did not find the *rib* gene in any of these *Proteus* O antigen gene clusters, indicating that the *rib* gene may be located outside the clusters in these strains.

There are 39 *Proteus* O antigens whose structures do not contain any rare monosaccharides (O3ab, O5, O9-O11, O13-O14, O16, O18, O20-O24, O26-O30, O33-O34, O37ab, O40-O41, O44, O47-O50, O52, O54ab, O57, O60-O61, O65, O69, and O71-O74). Of these, there are 38 antigens whose O antigen gene clusters are relatively simple and do not contain any genes responsible for the synthesis of rare monosaccharides. The only exception is O37ab, which is discussed below.

There are also some other genes in the O antigen gene clusters. Most O antigens of *Proteus* contain uronic acids, and the *ugd* and *gla* genes were found in many *Proteus* O antigen gene clusters [3]. The *ugd* gene is involved in UDP-GlcA biosynthesis [55, 56], and it was present in the O antigen gene clusters of all 60 O antigens (O56 has two copies of *ugd* gene). The *gla* gene, which is involved in UDP-GalA biosynthesis [55, 56], was found in 15 of the *Proteus* O antigen gene clusters. Of these, 14 O antigens (except for O61) contain GalA. The *gne* gene, which is responsible for UDP-GalNAc synthesis, was found in 12 *Proteus* O antigen gene clusters, and all corresponding O antigens contain GalNAc [57]. The *glf* gene, which is involved in the synthesis of UDP-Gal*f*, was found in 5 *Proteus* O antigen gene clusters, but none of these O antigens contain Gal*f*, suggesting that *glf* may not be involved in *Proteus* O antigen biosynthesis [58].

There are 3 *Proteus* O antigens (5% of the collection) whose O antigen gene clusters did not correspond to their known O antigen structures (O37ab, O53, and O55). The O37ab gene cluster contains *rmlA-qdtAB*, but its O antigen does not contain Qui3NAc. O53 antigen contains FucNAc but no *fnlABC* genes were found in the O antigen gene cluster. Similarly, O55 contains Rha in the O antigen but no *rmlACD* genes in the O antigen gene cluster. We have re-checked these three strains by sequencing based on serotype-specific genes, and the possibility of mixing up of other strains can be excluded. The possible explanation is that the strains we used in sequencing are different from those used for structure analysis. We will identify the O antigen structures of the three strains we had, and sequence the O antigen gene clusters of other strains of these three serotypes in the future.

### Glycosyltransferase genes

In *E*. *coli* and *Shigella*, the first sugar residue of the O antigen synthesis is GalNAc or GlcNAc, and the initial transferase (IT) encoded by *wecA* is responsible for initiating the O antigen synthesis, which is usually located outside the O antigen gene cluster [27, 59]. Almost all of the *Proteus* OPS structures analyzed in this study (except for O53) contain GalNAc or GlcNAc [32]. The IT genes are usually conserved across different species, and we identified the WecA in all 68 *Proteus* genomes, which had amino acid sequence identities to the homolog in *E*. *coli* K12 from 73.3% to 76.74% (S6 Table). The identities between the *Proteus* WecA are 90.46%-100% (data not shown). Therefore, we propose that WecA initiates the synthesis of the OPS in most *Proteus* strains.

Glycosyltransferases sequentially transfer sugars to growing glycan chains until the O-units have been completely synthesized [19, 21]. Each studied *Proteus* O antigen gene cluster contained 2 to 7 putative GT genes, with a total of 216 GT genes identified in the 60 O antigen gene clusters. According to the similarity of the protein sequences, we have classified 78 of these GTs into 19 homology groups that contain at least 2 GTs (HG01-HG19), as shown in S7 Table. The GTs in the same HGs are considered to have similar functions. By comparing the structures of the different O antigens that contain GTs belonging to the same HG, we predicted the functions of some of these GTs, and some examples are discussed below.

For instance, the GTs of HG02 share 62–65% identity to *E*. *coli* WbuB, which is a known L-FucNAc transferase [60]. By comparing the O antigen structures whose corresponding gene clusters contain GTs belonging to HG02, we found that O6, O8, O12, O19, and O42 all contain an α-L-FucNAc-(1→3)-D-GlcNAc linkage in their structures. Therefore, we predicted that the GTs of HG02 have a similar function and are responsible for the formation of the α-L-FucNAc-(1→3)-D-GlcNAc linkage.

In the same manner, the GTs of HG15 share 55% identity to *Citrobacter europaeus* PglA, which is a known α-1,3-D-GalNAc transferase [35, 61]. By comparing the known O antigen structures whose corresponding gene clusters contain GTs belonging to HG15 (O16 and O48), we found that they contain an α-D-GalNAc-(1→3)-D-GlcNAc linkage. Therefore, we predicted that the GTs of HG15 have a similar function and are responsible for the formation of the α-D-GalNAc-(1→3)-D-GlcNAc linkage.

By comparing the known 6 *Proteus* O antigen structures whose corresponding gene clusters containing GTs belonging to HG05, we found that they contain only one common linkage, α-D-GalA-(1→3) -D-GlcNAc. Therefore, we suggest that GTs of HG05 are responsible for the formation of the α-D-GalA-(1→3)-D-GlcNAc linkage.

### O antigen processing genes

**Fig 3:**
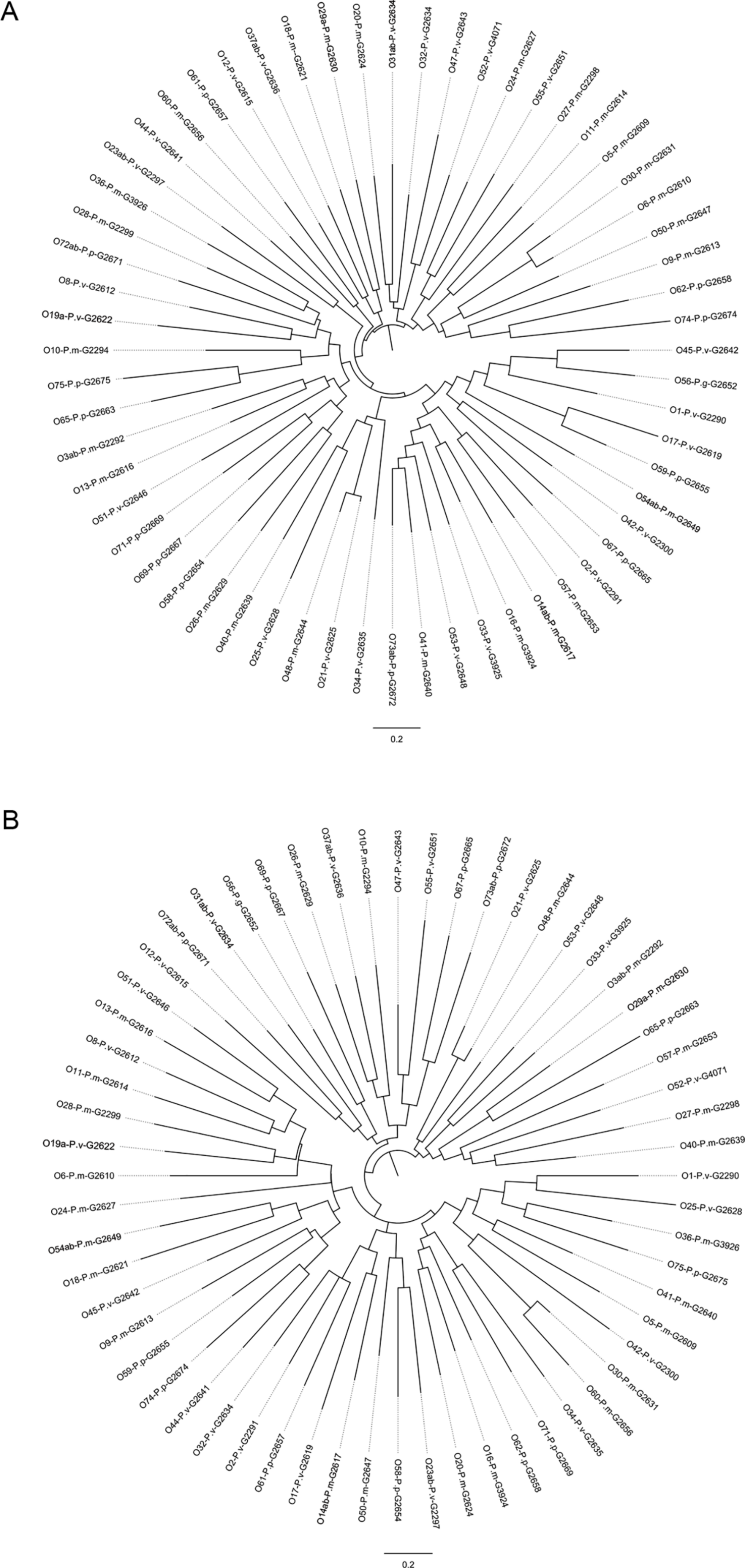
The phylogenetic trees for *wzx* and *wzy* genes from the 60 *Proteus* serotypes. The *wzx* (A) and *wzy* (B) trees were constructed using *wzx* and *wzy* genes. The sequences were aligned using ClustalW v2.0, and the trees were constructed using the JC69 substitution model and the phyML v3.0.

All 60 studied *Proteus* O antigen gene clusters contained both *wzx* and *wzy* genes, but none of them contained *wzz* gene. We identified the Wzz in all 68 *Proteus* genomes, which had amino acid sequence identities from 86.16% to 100% to *E*. *coli* K12 Wzz (S8 Table). Therefore, the *wzz* gene is located outside the *Proteus* O antigen gene clusters. As expected, all Wzx contain 10 to 12 transmembrane segments, and all Wzy contain 9 to 12 transmembrane segments. We constructed the maximum likelihood phylogenetic trees using the *wzx* and *wzy* genes, individually, which show the high levels of diversity of these two genes from different strains (Fig 3). The maximum gene sequence identity of *wzx* is 87.9%, and the maximum gene sequence identity of *wzy* is 82.7%; the identities between either *wzx* or *wzy* are not more than 80%. The diversity of the *wzx* and *wzy* genes provided us a basis to develop molecular techniques to ditect and identify different *Proteus* O serotypes.

### Additional genes identified

A putative methyltransferase gene, a glycerol-3-phosphate dehydrogenase gene, and two serine acetyltransferase genes were found between *cpxA* and *secB* genes in all 60 serotypes. The methyltransferase gene shared the same transcriptional promoter on the leading strand with the other O antigen synthesis genes. However, the other three genes use a different transcriptional promoter on the lagging strand, suggesting that these genes may not be associated with *Proteus* O antigen synthesis.

### PCR-based suspension arrays for molecular detection of 31 different *Proteus* O serotypes

**Fig 4:**
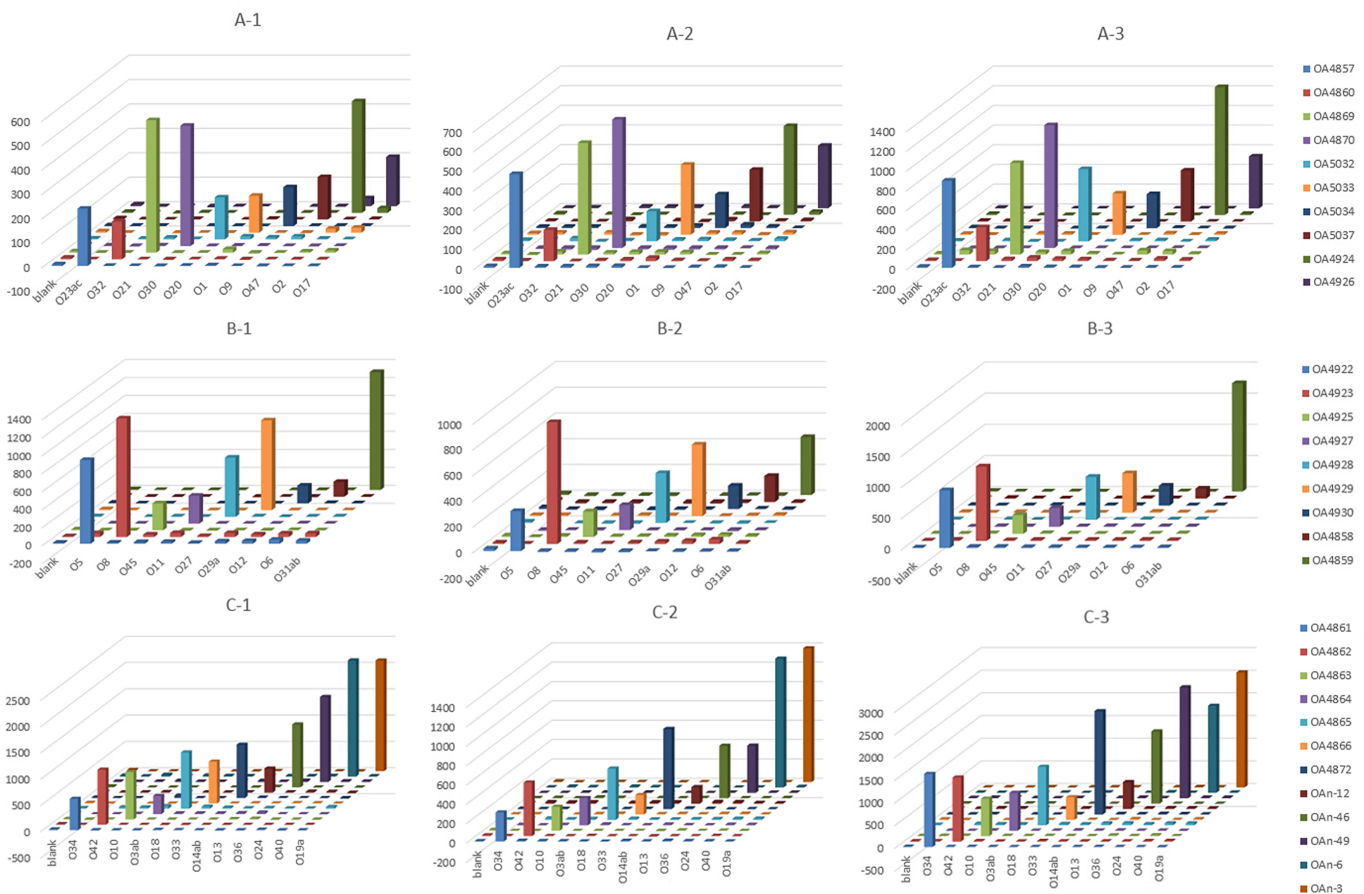
The hybridization results of the 31 *Proteus* strains. The suspension arrays were divided into 3 groups: (A) O1, O2, O9, O17, O20, O21, O23ac, O30, O32 and O47; (B) O5, O6, O8, O11, O12, O27, O29a, O31ab and O45; (C) O3ab, O10, O13, O14ab, O18, O19a, O24, O33, O34, O36, O40 and O42; no cross reactions were observed for any probe tested in this study, and the Blank was a negative control; the x-axis represents the PCR products of different serotypes, the y-axis represents the MFI values, and the z-axis represents the specific probes used for detection.

With the development of molecular techniques, many PCR-based molecular serotyping methods have been developed based on the O antigen specific genes for serological identification of many species, such as *E*. *coli*, *Salmonella* and *Yersinia pseudotuberculosis* [62]. According to the *Proteus* O antigen gene cluster analysis we performed in this study, the *wzx* and *wzy* genes were specific for different serotypes, indicating that the *wzx* and *wzy* genes could be used for molecular serotyping. At present, 37 *Proteus* serotypes have been reported to be frequently isolated from clinical specimens (O1-O15, O17-O21, O23-O24, O27-O34, O36, O38, O40, O42, and O45-O47) [35, 63, 64], and 31 of these 37 O antigens were analyzed in this study. A PCR-based suspension array was developed for molecular serotyping of all these 31 *Proteus* O serotypes using the *wzx* or *wzy* genes (Fig 4).

Primers were designed based on the *wzx* or *wzy* genes to amplify the PCR products, as described in the materials and methods. In most cases, we used *wzy* gene as target to amplify PCR products. If serotypes appeared to cross react due to the high sequence identities of their *wzy* genes, we tried to use *wzx* to obtain the PCR amplicons. Under optimal conditions, the multiplex PCR was performed to amplify the target amplicons varied from 100 to 495 bp. Serotype-specific probes (19 to 30 bp) were designed based on target genes for each serotype, and the optimum hybridization temperatures were determined by detecting the hybridization efficiencies at different temperatures (from 45°C to 60°C). Consequently, the probe hybridization temperature was determined to be 56–59°C.

To distinguish all of the 31 different strains tested, suspension arrays were divided into 3 groups: (A) O1, O2, O9, O17, O20, O21, O23ac, O30, O32 and O47; (B) O5, O6, O8, O11, O12, O27, O29a, O31ab and O45; (C) O3ab, O10, O13, O14ab, O18, O19a, O24, O33, O34, O36, O40 and O42. The results of the suspension array can be repeated from three repeat detections. The MFIs for probes hybridized with their homologous DNA are > 151.5, and the S/Bs for probes hybridized with their homologous DNA are > 14.9. The MFIs for probes hybridized with their nonhomologous DNA are < 51, and S/Bs for probes hybridized with their nonhomologous DNA are < 5.0. The MFIs and S/Bs for probes hybridized with their homologous DNA were significantly higher than the MFIs and S/Bs obtained from probes hybridized with nonhomologous DNA. No cross reactions were observed for any probe tested in this study (Fig 4).

In order to determine the sensitivity of the suspension array, a ten-fold gradient dilution experiment was carried out (0 fg/μl, 1.0 fg/μl, 10.0 fg/μl, 100.0 fg/μl, 1.0 pg/μl, 10.0 pg/μl, 100.0 pg/μl, 1.0 ng/μl and 10.0 ng/μl of genomic DNA). Positive signals could be generated as low as 10–100 pg of genomic DNA.

In conclusion, the primers and probes designed in this study worked well for each strain, and no obvious nonspecific signals were observed. However, like any other molecular detection method, this suspension array has limitations because the probes must be designed based on known sequences. More serotypes can be distinguished using this method if new specific probes and primers are designed to complement our suspension assay. Overall, this *wzx*/*wzy*-based suspension array provides us a potential tool to identify different *Proteus* O serotypes.

## Conclusions

OPS is an important component of gram-negative bacterial cell membranes with high variability within and between species. In this study, we identified 55 new O antigen gene clusters from different *Proteus* serotypes. Together with previously reported gene cluster data [35], we have analyzed a total of 60 *Proteus* O antigen gene clusters and have confirmed that the *Proteus* O antigen gene clusters are located between *cpxA* and *secB* genes, and the synthesis of *Proteus* O antigen is Wzx/Wzy pathway dependent. By comparison with their known O antigen structures, we found that most O antigen gene clusters correlated well with the corresponding O antigen structures (57 of 60, 95%). We also predicted the functions of some of the GTs by comparing the known O antigen structures whose corresponding gene clusters contain GTs belonging to the same HGs. The diversity of the *wzx* and *wzy* genes provides a basis for rapid molecular detection of different *Proteus* O serotypes. We therefore developed a suspension array to distinguish 31 different *Proteus* O serotypes using specific primers and probes designed based on the *wzx*/*wzy* genes. Our work comprehensively describes the O antigen gene clusters of *Proteus* species and provides a basis for future serological studies.

## Supporting information

S1 Table The strains used in this study (coming from the University of Lodz). ATCC–American Type Culture Collection, USA;
CCUG–Cultures Collection, University of Goeteborg, Sweden;
CDC–Center for Disease Control and Prevention, Atlanta, USA;
CNCTC–Czech National Collection of Type Cultures, Prague, Czech Republic;
NCTC–National Collection of Type Culture, London, UK.
(DOC)

S2 Table The primers used in this study. (DOC)

S3 Table The probe used in this study. (DOC)

S4 Table The O antigen structures from the 60 *Proteus* serotypes. (DOC)

S5 Table The 68 *Proteus* genomes. (DOC)

S6 Table The WecA in the 68 *Proteus* genomes. The amino acid sequence identities were obtained in comparison with the *E*. *coli* K12 WecA.
(DOC)

S7 Table The GTs and HGs. (DOC)

S8 Table The Wzz in the 68 *Proteus* genomes. The amino acid sequence identities were obtained in comparison with the *E*. *coli* K12 Wzz.
(DOC)
