# Supplementary material for: Genetic diversity of the O antigens of Proteus species and the development of a suspension array for molecular serotyping
Source: PLoS One. 2017 Aug 17;12(8):e0183267. doi: 10.1371/journal.pone.0183267 (PMC5560731; doi:10.1371/journal.pone.0183267)
Supplement: S1 Table — ATCC–American Type Culture Collection, USA; CCUG–Cultures Collection, University of Goeteborg, Sweden; CDC–Center for Disease Control and Prevention, Atlanta, USA; CNCTC–Czech National Collection of Type Cultures, Prague, Czech Republic; NCTC–National Collection of Type Culture, London, UK. (DOC) [file pone.0183267.s001.doc]

**S1 Table. The strains used in this study (coming from the University of Lodz).**

ATCC – American Type Culture Collection, USA

CCUG – Cultures Collection, University of Goeteborg, Sweden

CDC – Center for Disease Control and Prevention, Atlanta, USA

CNCTC – Czech National Collection of Type Cultures, Prague, Czech Republic

NCTC – National Collection of Type Culture, London, UK

| **Serotype** | **Lab. Collection Number** | **Species** | **Sources** | **Accession Number** |
| --- | --- | --- | --- | --- |
| O1 | G2290 | *Proteus vulgaris* | CCUG | KY710685 |
| O2 | G2291 | *Proteus vulgaris* | CNCTC | KY710686 |
| O3ab | G2292 | *Proteus mirabilis* | CCUG | GU254059 |
| O5 | G2609 | *Proteus mirabilis* | CNCTC | KY710687 |
| O6 | G2610 | *Proteus mirabilis* | CNCTC | KY710688 |
| O8 | G2612 | *Proteus vulgaris* | CNCTC | KY710689 |
| O9 | G2613 | *Proteus mirabilis* | CNCTC | KY710690 |
| O10 | G2294 | *Proteus mirabilis* | CNCTC | GU254060 |
| O11 | G2614 | *Proteus mirabilis* | CNCTC | KY710691 |
| O12 | G2615 | *Proteus vulgaris* | CNCTC | KY710692 |
| O13 | G2616 | *Proteus mirabilis* | CNCTC | KY710693 |
| O14ab | G2617 | *Proteus mirabilis* | CNCTC | KY710694 |
| O16 | G3924 | *Proteus mirabilis* | CNCTC | KY710695 |
| O17 | G2619 | *Proteus vulgaris* | CCUG | KY710696 |
| O18 | G2621 | *Proteus mirabilis* | CNCTC | KY710697 |
| O19a | G2622 | *Proteus vulgaris* | CNCTC | KY710698 |
| O20 | G2624 | *Proteus mirabilis* | CNCTC | KY710699 |
| O21 | G2625 | *Proteus vulgaris* | CNCTC | KY710700 |
| O23ac | G2297 | *Proteus vulgaris* | CCUG | GU254061 |
| O24 | G2627 | *Proteus mirabilis* | CNCTC | KY710701 |
| O25 | G2628 | *Proteus vulgaris* | CNCTC | KY710702 |
| O26 | G2629 | *Proteus mirabilis* | CNCTC | KY710703 |
| O27 | G2298 | *Proteus mirabilis* | CNCTC | GU254062 |
| O28 | G2299 | *Proteus mirabilis* | CNCTC | KY710738 |
| O29a | G2630 | *Proteus mirabilis* | CNCTC | KY710704 |
| O30 | G2631 | *Proteus mirabilis* | CNCTC | KY710705 |
| O31ab | G2632 | *Proteus vulgaris* | CNCTC | KY710706 |
| O32 | G2634 | *Proteus vulgaris* | CNCTC | KY710707 |
| O33 | G3925 | *Proteus mirabilis* | CNCTC | KY710708 |
| O34 | G2635 | *Proteus vulgaris* | CCUG | KY710709 |
| O36 | G3926 | *Proteus mirabilis* | CNCTC | KY710710 |
| O37ab | G2636 | *Proteus vulgaris* | CNCTC | KY710711 |
| O40 | G2639 | *Proteus mirabilis* | CNCTC | KY710712 |
| O41 | G2640 | *Proteus mirabilis* | CNCTC | KY710713 |
| O42 | G2300 | *Proteus vulgaris* | CCUG | KY710714 |
| O44 | G2641 | *Proteus vulgaris* | CNCTC | KY710715 |
| O45 | G2642 | *Proteus vulgaris* | CCUG | KY710716 |
| O47 | G2643 | *Proteus vulgaris* | CNCTC | GU254063 |
| O48 | G2644 | *Proteus mirabilis* | CNCTC | KY710717 |
| O50 | G2647 | *Proteus mirabilis* | University of Toronto, Canada | KY710718 |
| O51 | G2646 | *Proteus mirabilis* | CCUG | KY710719 |
| O52 | G4071 | *Proteus vulgaris* | ATCC | KY710720 |
| O53 | G2648 | *Proteus vulgaris* | University of Toronto, Canada | KY710721 |
| O54ab | G2650 | *Proteus mirabilis* | CCUG | KY710722 |
| O55 | G2651 | *Proteus vulgaris* | University of Toronto, Canada | KY710723 |
| O56 | G2652 | *Proteus* genomospecies 4 | CDC | KY710724 |
| O57 | G2653 | *Proteus mirabilis* | University of Toronto, Canada | KY710725 |
| O58 | G2654 | *Proteus penneri* | CDC | KY710726 |
| O59 | G2655 | *Proteus penneri* | CDC | KY710727 |
| O60 | G2656 | *Proteus myxofaciens* | ATCC | KY710739 |
| O61 | G2657 | *Proteus penneri* | CDC | KY710728 |
| O62 | G2658 | *Proteus penneri* | CDC | KY710729 |
| O65 | G2663 | *Proteus penneri* | CDC | KY710730 |
| O67 | G2665 | *Proteus penneri* | CDC | KY710731 |
| O69 | G2667 | *Proteus penneri* | CDC | KY710732 |
| O71 | G2669 | *Proteus penneri* | CDC | KY710733 |
| O72ab | G2671 | *Proteus penneri* | CDC | KY710734 |
| O73ab | G2672 | *Proteus penneri* | NCTC | KY710735 |
| O74 | G2674 | *Proteus mirabilis* | CCUG | KY710736 |
| O75 | G2675 | *Proteus mirabilis* | CCUG | KY710737 |
