# Supplementary material for: Genetic diversity of the O antigens of Proteus species and the development of a suspension array for molecular serotyping
Source: PLoS One. 2017 Aug 17;12(8):e0183267. doi: 10.1371/journal.pone.0183267 (PMC5560731; doi:10.1371/journal.pone.0183267)
Supplement: S2 Table — (DOC) [file pone.0183267.s002.doc]

**S2 Table. The primers used in this study.**

| **Serotype** | **Forward or Reverse** | **Lab. Primer Number** | **Primer** | **TM Value** |
| --- | --- | --- | --- | --- |
| O1 | Forward | wl70823 | 5' TAATTACTAGTGTTTTAATCTCACT 3' | 48.4 |
|  | Reverse | wl70824 | 5' TAAACCGAGTAGCAAAGTAAA 3' | 50.9 |
| O2 | Forward | wl70533 | 5' ATTTATCTCCATTCCCATTC 3' | 50.8 |
|  | Reverse | wl70534 | 5' AACCCATAGGACCACTCAT 3' | 50.9 |
| O3ab | Forward | wl69883 | 5' ATTCTCTGGGCTCAGATATG 3' | 51.5 |
|  | Reverse | wl69884 | 5' AATATAGCAAGTAGATGATAAAAGA 3' | 50.8 |
| O5 | Forward | wl69851 | 5' TTAGCCATAGGAGTAGTTTTCTT 3' | 52.8 |
|  | Reverse | wl69852 | 5' TTCAGGAACTTTAGTGGAATATA 3' | 51.6 |
| O6 | Forward | wl69867 | 5' TATTGGTGGTACTATACAATTCTT 3' | 51.1 |
|  | Reverse | wl69868 | 5' AGATATATTTCCTTCTAGAGTTGTT 3' | 51 |
| O8 | Forward | wl69855 | 5' GCAGGCCCAATAGGAGA 3' | 53.4 |
|  | Reverse | wl69856 | 5' ATCTGAAGCAAATCCACGTA 3' | 53.1 |
| O9 | Forward | wl70825 | 5' TAGTCAATCTAATTGAGCGTG 3' | 50.9 |
|  | Reverse | wl70826 | 5' ATAAGAAGGGATGTTGTAATT 3' | 48.2 |
| O10 | Forward | wl69879 | 5' CTGTAGGTACTGTAGTTGATGTAATT 3' | 52.6 |
|  | Reverse | wi69880 | 5' ATAACACCATAGCTAGAGAATGC 3' | 53.4 |
| O11 | Forward | wl69859 | 5' CTCTTATTAATACCTATTATATATCTCTCT 3' | 51.9 |
|  | Reverse | wl69860 | 5' TATTGGTAAGCTAATTATTATGG 3' | 50.1 |
| O12 | Forward | wl69865 | 5' TAGCTTTCTTGATATAATCATTAAG 3' | 51.4 |
|  | Reverse | wl69866 | 5' TCCTAATTTGCTAAATATGCTAG 3' | 52.3 |
| O13 | Forward | wln-233 | 5' ATCAAAAAAAATTGAGCTTT 3' | 49.5 |
|  | Reverse | wln-234 | 5' CATAGTCCATGTAGATAAAAAAA 3' | 49.9 |
| O14ab | Forward | wl70541 | 5' ATTACTTACGGAAGAACCTTTA 3' | 51 |
|  | Reverse | wl70542 | 5' ACTAGTCGCTAGGGATTGTC 3' | 51.1 |
| O17 | Forward | wl70537 | 5' TTATACAGAGGGCATCGC 3' | 51.3 |
|  | Reverse | wl70538 | 5' TATGGGAGTGTTTTCTGGTA 3' | 50.4 |
| O18 | Forward | wl70527 | 5' CAAGCTTGGTTCTGGGT 3' | 50.3 |
|  | Reverse | wl70528 | 5' ATCGGTGGAATCCTATTGA 3' | 52.3 |
| O19a | Forward | wln-215 | 5' ATTGCTATCTTAGGTTTCTTATTA 3' | 50.8 |
|  | Reverse | wln-216 | 5' TTTATGATTAACGCAGCATAC 3' | 51.6 |
| O20 | Forward | wl70821 | 5' TGTGCTTTCTTCAATATTATT 3' | 52 |
|  | Reverse | wl70822 | 5' TTGTAATTTGCTGGTTGGTT 3' | 53.4 |
| O21 | Forward | wl69871 | 5' TATGCAGACAACGAGTGCT 3' | 51.6 |
|  | Reverse | wl69872 | 5' AAAGATAGCAAGCCACTCA 3' | 50.5 |
| O23ac | Forward | wl69847 | 5' TTTAGATTTACCGTTTTCTGGT 3' | 50 |
|  | Reverse | wl69848 | 5' ACCAAAGTTCAGTAAGGCTTCT 3' | 54 |
| O24 | Forward | wln-239 | 5' GCTTGTAGCTTTGGCTTAG 3' | 50.8 |
|  | Reverse | wln-240 | 5' TTGCGGATGATATGTTGA 3' | 50 |
| O27 | Forward | wl69861 | 5' ATACTAAGGCAAGGTCTAGCA 3' | 51.9 |
|  | Reverse | wl69862 | 5' ATAATATAAGTCCTTATCCTAAACTTA 3' | 51.3 |
| O29a | Forward | wl69863 | 5' GATGCCATCACTCGGTATA 3' | 50.9 |
|  | Reverse | wl69864 | 5' GAATTATCCCAATAGCACCTA 3' | 51.9 |
| O30 | Forward | wl69873 | 5' TTAGAAGATGATAGGTTTGGAG 3' | 51.3 |
|  | Reverse | wl69874 | 5' AAAGGGCTGTTACTGTGAAT 3' | 51.5 |
| O31ab | Forward | wl69869 | 5' TTGATAGGACTAATTAGTATTGCA 3' | 52.2 |
|  | Reverse | wl69870 | 5' TTAATTGCACTTATAGATGTGAA 3' | 50.9 |
| O32 | Forward | wl69853 | 5' AAAACACAATATAGCAATATCT 3' | 46 |
|  | Reverse | wl69854 | 5' TTATTTGGTGAGTGCTTTTTCT 3' | 51 |
| O33 | Forward | wl70529 | 5' AATACGATTACTCAAGAGATAAACA 3' | 53 |
|  | Reverse | wl70530 | 5' AATAGAGGTTCTTGCAGACAA 3' | 52 |
| O34 | Forward | wl69875 | 5' CACTTTCAAGTATCGCAATAATA 3' | 53.1 |
|  | Reverse | wl69876 | 5' AGCTATTATGACAGCCCAATAT 3' | 53.5 |
| O36 | Forward | wln-790 | 5' TCTATTACCCAGAACTAAAAAG 3' | 49.1 |
|  | Reverse | wln-791 | 5' CAACCACCACCCACC 3' | 48.1 |
| O40 | Forward | wln-221 | 5' TGGGGCAATGACTTCAG 3' | 51.8 |
|  | Reverse | wln-222 | 5' TCATTTTCTCATGGGCA 3' | 49.4 |
| O42 | Forward | wl69877 | 5' GGTATACACGAAAAGGTAATAGA 3' | 51.4 |
|  | Reverse | wl69878 | 5' AACCCTGATAGAGAGTCAAGAA 3' | 52.7 |
| O45 | Forward | wl69857 | 5' AAATAATGTACTGGATTACAAGTT 3' | 50.5 |
|  | Reverse | wl69858 | 5' AGACAAAACCCGGTTCATA 3' | 52.7 |
| O47 | Forward | wl70831 | 5' CATCAGTTCAAGGTCGGCT 3' | 55.5 |
|  | Reverse | wl70832 | 5' CCCCACCGAATACCAAAT 3' | 55.2 |
