# Supplementary material for: Genetic diversity of the O antigens of Proteus species and the development of a suspension array for molecular serotyping
Source: PLoS One. 2017 Aug 17;12(8):e0183267. doi: 10.1371/journal.pone.0183267 (PMC5560731; doi:10.1371/journal.pone.0183267)
Supplement: S3 Table — (DOC) [file pone.0183267.s003.doc]

**S3 Table. The probe used in this study.**

| **Serotype** | **Lab. Probe Number** | **Probe** | **TM Value** |
| --- | --- | --- | --- |
| O1 | OA5033 | 5' GTTGAAATATCAGGGCTAAG 3' | 56.1 |
| O2 | OA4924 | 5' GAAATAAAATGAATGGGAATGG 3' | 56.2 |
| O3ab | OA4864 | 5' GCTCAGATATGATGCTGGAAATGAT 3' | 61.8 |
| O5 | OA4922 | 5' ATCTGGTGATTTGACTAGTGGAAG 3' | 57.3 |
| O6 | OA4858 | 5' AACTAGCCCTGGACGTGGC 3' | 59.8 |
| O8 | OA4923 | 5' ACGTGGTGTAATGAAAGGTGGT 3' | 58.6 |
| O9 | OA5034 | 5' TTGAGCGTGCTTCTG 3' | 56.4 |
| O10 | OA4863 | 5' TTGGCTGGAGGAGGAGGAGTT 3' | 62.5 |
| O11 | OA4927 | 5' GCAGGTAGTAGAGCGGCAATAG 3' | 59.1 |
| O12 | OA4930 | 5' AAGTTAGGCGTTGACTGGCTCT 3' | 60.2 |
| O13 | OAn-12 | 5' TCAAGGAATGGGAAAAACTAAAAT 3' | 58.9 |
| O14ab | OA4872 | 5' TGAGTTATTACCCCACGTTTATGTT 3' | 59.5 |
| O17 | OA4926 | 5' TTTAAGCGATGCCCTCTGTAT 3' | 57.2 |
| O18 | OA4865 | 5' GTTCTGGGTTTGGCGTAATACTAA 3' | 60.1 |
| O19a | OAn-3 | 5' ACAAGGGATAGACCAATTAATTCAAA 3' | 60.3 |
| O20 | OA5032 | 5' GAGTCGTTACTACTCAATTCACGGGC 3' | 59.8 |
| O21 | OA4869 | 5' GGAATTATCGCCCTAGTGGT 3' | 56.1 |
| O23ac | OA4857 | 5' CAAATGGCACAGTATATGGA 3' | 50 |
| O24 | OAn-49 | 5' ATCCTCTATAATTTTCATATATACTCAAGATA 3' | 57.3 |
| O27 | OA4928 | 5' CCATTCGCCCTTATCAGCAT 3' | 60.1 |
| O29 | OA4929 | 5' TCCTTTGGTCTGTAAGCTACAGTAA 3' | 57.9 |
| O30 | OA4870 | 5' AGGTTTGGAGAAAGGTTTACAG 3' | 58.2 |
| O31ab | OA4859 | 5' CGAAGTAACTCGGAGCTTAGTCTAGA 3' | 60.6 |
| O32 | OA4860 | 5' ATTAGCCCCACAAGTGGAAA 3' | 56.3 |
| O33 | OA4866 | 5' TGCACTCTGATGGAGTAAGAGGAA 3' | 60.8 |
| O34 | OA4861 | 5' TGTTTTGGGTAAGTGGGCAGAT 3' | 61.2 |
| O36 | OAn-46 | 5' CCAGAACTAAAAAGAATGTTTATGAA 3' | 57.2 |
| O40 | OAn-6 | 5' GGTCCAGATAGAATCAAAGCTGC 3' | 60 |
| O42 | OA4862 | 5' GCAACTGCTTCCTCCGTTATTG 3' | 61.7 |
| O45 | OA4925 | 5' AATAATACGACTTTCTGCGTCTTT 3' | 57.2 |
| O47 | OA5037 | 5' AATCCATTAGCTGGCGAA 3' | 56 |
