# Supplementary material for: Genetic diversity of the O antigens of Proteus species and the development of a suspension array for molecular serotyping
Source: PLoS One. 2017 Aug 17;12(8):e0183267. doi: 10.1371/journal.pone.0183267 (PMC5560731; doi:10.1371/journal.pone.0183267)
Supplement: S4 Table — (DOC) [file pone.0183267.s004.doc]

**S4 Table. The O antigen structures from the 60 *Proteus* serotypes [1].**

| **Serotype** | **O antigen structure** |
| --- | --- |
| O1 | l-Qui*p*NAc-(α1↓  3)  →4)-d-Gal*p*NAc-(α1→4)-d-Gal*p*-1-*P*-(O→4)-l-Qui*p*NAc-(α1→3)-d-Glc*p*NAc-(β1→ |
| O2 | →2)-d-Glc*p*-(β1→6)-d-Glc*p*NAc-(α1→3)-l-Qui*p*NAc-(α1→3)-Glc*p*NAc6Ac-(β1→ |
| O3ab | d-Gal*p*A6(l-lys)-(α1↓ d-Glc*p*-(α1↓  4) 2)  →6)-d-Gal*p*NAc-(β1→4)-d-Glc*p*A-(β1→3)-d-Gal*p*NAc-(β1→ |
| O5 | →4)-d-Glc*p*NAc3,6Ac-(α1→4)-d-Gal*p*A-(α1→3)-d-Gal*p*A-(α1→3)-d-Glc*p*NAc-(β1→ |
| O6 | d-Glc*p*A-(α1↓  3)  →4)-l-Fuc*p*NAc-(α1→3)-d-Glc*p*NAc-(β1→ |
| O8 | d-Gal*p*-(α1↓  3)  →3)-d-Glc*p*A-(β1→4)-l-Fuc*p*NAc-(α1→3)-d-Glc*p*NAc-(α1→ |
| O9 | →4)-d-Gal*p*A-(α1→2)-d-Rib*f*3Ac-(β1→4)-d-Gal*p*-(β1→3)-d-Glc*p*NAc-(α1→ |
| O10 | l-Alt*p*A-(α1↓  3)  →4)-d-Gal*p*NAc-(α1→3)-d-Gal*p*A-(α1→3)-d-Glc*p*NAc-(α1→ |
| O11 | d-Glc*p*NAc-(α1↓ d-Glc*p*-(α1↓  2) 6)  →4)-d-Glc*p*A-(β1→3)-d-Gal*p*A6(l-Thr)-(β1→3)-d-Glc*p*NAc-(β1→ |
| O12 | d-Glc*p*-(α1→6)-d-Gal*p*NAc4Ac-(α1↓  3)  →3)-d-Gro-1-*P*-(O→6)-d-Glc*p*-(β1→4)-l-Fuc*p*NAc-(α1→3)-d-Glc*p*NAc-(β1→ |
| O13 | d-Gal*p*A6(*R*-Cet-l-lys)-(α1↓  4)  →3)- d-Gal*p*-(α1→3)-d-Glc*p*NAc-(β1→ |
| O14ab | *R*-Cet-Etn*P*↓  6  →4)-d-Gal*p*-(α1→4)-d-Gal*p*NAc-(β1→3)-d-Gal*p*6Ac-(α1→3)- d-Gal*p*NAc-(β1→ |
| O16 | Etn*P*↓  6  →2)-d-Rib-ol-5-*P*-(O→6)-d-Gal*p*NAc-(β1→4)-d-Gal*p*NAc-(α1→3)-d-Glc*p*NAc-(α1→ |
| O17 | →2)-d-Fuc*p*3N(*R*-3HOBu)-(β1→6)-d-Glc*p*3Ac-(α1→4)-d-Glc*p*A-(β1→3)-d-Glc*p*NAc-(α1→ |
| O18 | Cho*P*↓ d-Glc*p*-(α1↓  4 4)  →3)-d-Glc*p*NAc-1-*P*-(O→6)-d-Glc*p*-(β1→3)-d-Gal*p*-(β1→3)-d-Glc*p*NAc-(β1→ |
| O19a | →3)-d-Gal*p*-(α1→4)-d-Gal*p*NAc-(α1→3)-l-Fuc*p*NAc-(α1→3)-d-Glc*p*NAc-(β1→ |
| O20 | d-Glc*p*-(α1→2)-d-Gal*p*-(β1↓  4)  →3)-d-Glc*p*NAc-(α1→4)-d-Glc*p*-(β1→3)-d-Glc*p*NAc-(β1→ |
| O21 | d-Glc*p*-(α1↓  6)  →2)-d-Glc*p*-1-*P*-(O→6)-d-Glc*p*NAc-(α1→4)-d-Gal*p*NAc-(α1→3)-d-Gal*p*NAc-(β1→ |
| O23ac | →2)-d-Gal*p*A4Ac-(β1→3)-d-Gal*p*NAc-(α1→4)-d-Gal*p*A-(α1→3)-d-Glc*p*NAc6Ac-(β1→ |
| O24 | d-Gal*p*3,4(*S*-Pyr)-(β1↓  3  →4)-d-Gal*p*NAc-(β1→4)-d-Glc*p*NAc-(β1→3)-d-Glc*p*NAc-(β1→ |
| O25 | d-Glc*p*3(*R*-Lac)-(α1↓  3)  →2)-l-Rha*p*-(α1→2)-d-Rib*f*-(β1→4)-d-Gal*p*NAc-(α1→3)-d-Glc*p*NAc-(β1→ |
| O26 | →4)-d-Gal*p*A6(l-lys)-(α1→4)-d-Gal*p*A-(α1→3)-d-Gal*p*A4Ac-(β1→3)-d-Glc*p*NAc-(β1→ |
| O27 | d-Glc*p*NAc-(β↓ Etn*P*↓  4) 6  →3)-d-Glc*p*A6(l-lys)-(β1→3)-d-Gal*p*A6(l-Ala)-(α1→3)-d-Glc*p*NAc-(β1→ |
| O28 | →4)-d-Gal*p*A6(l-lys)-(α1→4)-d-Gal*p*A-(α1→3)-d-Gal*p*A6(l-Ser)4Ac-(α1→3)-d-Glc*p*NAc-(β1→ |
| O29a | d-Gal*p*NAc-(α1↓  3)  →4)-d-Gal*p*NAc-(β1→4)-d-Glc*p*A-(β1→3)-d-Gal*p*NAc-(β1→ |
| O30 | →4)-d-Glc*p*A-(β1→6)-d-Gal*p*NAc-(α1→6)-d-Glc*p*NAc-(β1→3)-d-Glc*p*NAc4Ac-(β1→ |
| O31ab | →6)-d-Glc*p*NAc3(*S*-lac)-(α1→3)-l-Qui*p*NAc-(α1→3)-d-Glc*p*NAc-(α1→ |
| O32 | →4)-d-Gal*p*A-(α1→2)-l-Rha*p*-(α1→2)-l-Rha*p*-(α1→4)-d-Gal*p*A-(β1→3)-d-Glc*p*NAc-(β1→ |
| O33 | d-Rib-ol-5-*P*-(O↓ Etn*P*↓  3) 6  →2)-d-Gal*p*-(β1→3)-d-Glc*p*NAc-(α1→3)-d-Glc*p*-(β1→3)-d-Glc*p*NAc-(β1→ |
| O34 | d-Glc*p*-(β1↓  2)  →4)-d-Gal*p*NAc-1-*P*-(O→6)-d-Gal*p*-(β1→3)-d-Gal*p*NAc-(β1→ |
| O36 | →2)-d-Rib*f*-(β1→4)-d-Gal*p*-(β1→4)-d-Glc*p*NAc6Ac-(α1→4)-d-Gal*p*-(β1→3)-d-Glc*p*NAc-(α1→ |
| O37ab | →3)-d-Glc*p*A-(β1→4)-d-Glc*p*-(α1→3)-d-Glc*p*A-(β1→3)-d-Glc*p*NAc6Ac-(α1→ |
| O40 | →3)-d-Glc*p*NAc4(*R*-Lac)-(β1→3)-d-Gal*p*-(α1→3)-d-Gro-1-*P*-(O→3)-d-Glc*p*NAc-(β1→ |
| O41 | d-Rib-ol-5-*P*-(O↓ Etn*P*↓ Etn*P*↓  6) 6 6  →3)-d-Gal*p*-(α1→6)-d-Glc*p*NAc-(β1→3)-d-Gal*p*-(β1→3)-d-Gal*p*NAc-(β1→ |
| O42 | →3)-l-Fuc*p*NAc-(α1→4)-d-Glc*p*-1-*P*-(O→4)-d-Glc*p*NAc-(α1→3)-l-Fuc*p*NAc-(α1→  3)-d-Glc*p*NAc‑(α1→ |
| O44 | →4)-d-Glc*p*-(β1→3)-d-Gal*p*-(α1→4)-d-Gal*p*NAc-(β1→4)-d-Glc*p*A(l-Ala)-(β1→3)-d-Gal*p*NAc-(β1→ |
| O45 | →2)-l-Fuc*p*3NAc-(β1→6)-d-Glc*p*NAc-(α1→4)-d-Gal*p*NAc-(α1→4)-d-Gal*p*A-(α1→  3)-d-Glc*p*NAc-(β1→ |
| O47 | d-Glc*p*A-(β1↓  4)  →3)-d-Gal*p*NAc-(β1→4)-d-Gal*p*NAc3Ac-(α1→3)-d-Gal*p*NAc-(β1→ |
| O48 | →2)-d-Gal*p*-1-*P*-(O→6)-d-Glc*p*NAc3Ac-(α1→4)-d-Gal*p*NAc-(α1→3)-d-Glc*p*NAc-(β1→ |
| O50 | d-Glc*p*-(β1↓  4)  →3)-d-Glc*p*A-(β1→4)-d-Gal*p*NAc-(β1→4)-d-Gal*p*-(β1→3)-d-Glc*p*NAc-(β1→ |
| O51 | →3)-d-Gal*p*NAc4,6(*R*-Pyr)-(α1→4)-d-Gal*p*A-(α1→3)-l-Rha*p*2Ac-(α1→3)-d-Glc*p*NAc-(β1→ |
| O52 | →2)-d-Gal*p*4,6(*R*-Pyr)-(α1→4)-d-Gal*p*-(β1→3)-d-Glc*p*NAc-(β1→ |
| O53 | →1)-d-Rib-ol-5-*P*-(O→1)-d-Rib-ol2/3/4Ac-5-*P*-(O→3)-d-Fuc*p*NAc4N-(β1→ |
| O54ab | d-Gro-1-*P*-(O↓  4)  →6)-d-Glc*p*NAc-(α1→3)-d-Gal*p*-(β1→3)-d-Gal*p*NAc-(α1→ |
| O55 | l-Rha*p*NAc-(α1↓  4)  →3)-d-Gal*p*NAcA-(α1→3)-l-Qui*p*NAc-(α1→4)-d-Glc*p*NAc-(α1→ |
| O56 | d-Glc*p*-(α1↓  2)  →4)-d-Qui*p*3NAc-(β1→6)-d-Glc*p*NAc-(β1→4)-d-Gal*p*A-(β1→3)-d-Gal*p*NAc-(α1→ |
| O57 | d-Gro-1-*P*-(O↓ d-Glc*p*-(α1↓  3) 6)  →4)-d-Gal*p*NAc-(β1→3)-d-Gal*p*-(α1→6)-d-Gal*p*-(β1→3)-d-Gal*p*NAc-(β1→ |
| O58 | d-Gal*p*A6(l-Thr)3Ac-(α1↓  3)  →4)-d-Gal*p*NAc-(β1→3)-l-Rha*p*-(β1→4)-d-Glc*p*NAc6Ac-(β1→ |
| O59 | →2)-d-Qui*p*3N(Ac-d-Ala)-(β1→4)-d-Gal*p*A6(l-Ala)-(α1→2)-d-Rib*f*-(β1→4)-d-Gal*p*-(β1→  3)-d-Glc*p*NAc-(β1→ |
| O60 | →4)-d-Glc*p*A6(*S*-Cet-l-lys)-(β1→6)-d-Gal*p*NAc-(α1→6)-d-Glc*p*NAc-(β1→3)-d-Glc*p*NAc-(β1→ |
| O61 | d-Glc*p*NAc-(β1↓  3)  →4)-d-Gal*p*NAc-(β1→3)-d-Gal*p*NAc-(α1→4)-d-Gal*p*A-(β1→3)-d-Glc*p*NAc-(β1→ |
| O62 | d-Glc*p*-(β1→3)-d-Glc*p*NAc4(*S*-Lac)-(β1↓  2)  →3)-l-Rha*p*-(α1→2)-l-Rha*p*-(α1→2)-d-Gal*p*6Ac-(α1→  3)-d-Glc*p*NAc-(β1→ |
| O65 | →4)-d-Gal*p*NAc-(β1→4)-d-Gal*p*-(β1→4)-d-Glc*p*-(β1→3)-d-Gal*p*NAc-(β1→ |
| O67 | d-Glc*p*-(α1↓ d-Glc*p*-(β1↓ Etn*P*↓  6) 4) 6  →4)-d-Gal*p*A-(β1→3)-d-Gal*p*NAc-(α1→3)-l-Fuc*p*NAc-(α1→3)-d-Glc*p*NAc-(β1→ |
| O69 | d-Glc*p*A3/4Ac-(α1↓  4)  →6)-d-Glc*p*N(l-Ala)3Ac-(β1→4)-d-Glc*p*A-(β1→3)-d-Glc*p*NAc6Ac-(β1→ |
| O71 | →4)-d-Gal*p*A-(α1→2)-d-Glc*p*-(β1→4)-d-Glc*p*-(β1→3)-d-Glc*p*NAc-(β1→ |
| O72ab | d-Gal*p*NAc6Ac-(β1↓ d-Glc*p*-(α1↓  3) 6)  →4)-d-Gal*p*-(α1→6)-d-Glc*p*-(β1→3)-d-Gal*p*NAc-(β1→ |
| O73ab | Etn*P*↓  6  →4)-d-Rib-ol-5-*P*-(O→4)-d-Glc*p*-(β1→3)-d-Gal*p*-(β1→3)-d-Gal*p*NAc-(β1→ |
| O74 | d-Glc*p*NAc-(α1↓  4)  →3)-d-Gal*p*A2Ac-(α1→3)-d-Gal*p*A6(l-Ala)-(α1→3)-d-Glc*p*NAc-(α1→ |
| O75 | d-Gal*p*NAc-(β1↓  4)  →3)-d-Gal*p*-(α1→4)-l-Rha*p*-(α1→3)-d-Glc*p*NAc-(β1→ |

**References:**

1. Knirel YA, Perepelov AV, Kondakova AN, Senchenkova SN, Sidorczyk Z, Rozalski A, et al. Structure and serology of O-antigens as the basis for classification of Proteus strains. Innate Immun. 2011;17(1):70-96. doi: 10.1177/1753425909360668. PubMed PMID: 20305038.
