# Supplementary material for: Genetic diversity of the O antigens of Proteus species and the development of a suspension array for molecular serotyping
Source: PLoS One. 2017 Aug 17;12(8):e0183267. doi: 10.1371/journal.pone.0183267 (PMC5560731; doi:10.1371/journal.pone.0183267)
Supplement: S5 Table — (DOC) [file pone.0183267.s005.doc]

**S5 Table. The 68 *Proteus*** genomes.

| **Genome ID** | **Strains** |
| --- | --- |
| FNAA01000018.1 | Proteus mirabilis strain NLAE-zl-C285 |
| CP020052.1 | Proteus mirabilis strain AR_0059 |
| MBTY01000036.1 | Proteus mirabilis strain T1C |
| LWUM01000125.1 | Proteus mirabilis strain PM_178 |
| LWUL01000070.1 | Proteus mirabilis strain PM_125 |
| CP015347.1 | Proteus mirabilis strain AOUC-001 |
| LWDB01000010.1 | Proteus mirabilis strain NIVEDI3-PG74 |
| LQNO01000021.1 | Proteus mirabilis strain GB11 |
| LQNN01000019.1 | Proteus mirabilis strain GB08 |
| KV388090.1 | Proteus mirabilis strain C02011 |
| KQ960990.1 | Proteus mirabilis strain GED7834 |
| LGAY01000041.1 | Proteus mirabilis strain NO-051/03 |
| CP012675.1 | Proteus vulgaris strain CYPV1 |
| CP012674.1 | Proteus mirabilis strain CYPM1 |
| JUYT01000059.1 | Proteus mirabilis strain 646_PMIR |
| JVHX01000049.1 | Proteus mirabilis strain 418_PMIR |
| JUXR01000308.1 | Proteus mirabilis strain 672_PMIR |
| JVMQ01000065.1 | Proteus mirabilis strain 292_PMIR |
| JVKD01000015.1 | Proteus mirabilis strain 360_PMIR |
| JVPB01000062.1 | Proteus mirabilis strain 232_PMIR |
| JVTJ01000130.1 | Proteus mirabilis strain 1330_PMIR |
| JVUE01000042.1 | Proteus mirabilis strain 1313_PMIR |
| JVXV01000113.1 | Proteus mirabilis strain 1230_SSON |
| JWAP01000093.1 | Proteus mirabilis strain 1166_PMIR |
| JVTO01000142.1 | Proteus mirabilis strain 1326_PMIR |
| JVVD01000056.1 | Proteus mirabilis strain 1293_PMIR |
| JWCS01000112.1 | Proteus mirabilis strain 1114_PMIR |
| JTBP01000001.1 | Proteus mirabilis strain FDAARGOS_67 |
| JTBB01000001.1 | Proteus mirabilis strain FDAARGOS_80 |
| JTBA01000001.1 | Proteus mirabilis strain FDAARGOS_81 |
| JTAW01000004.1 | Proteus mirabilis strain FDAARGOS_85 |
| JSCB01000061.1 | Proteus mirabilis strain Pm-Oxa48 |
| KN150749.1 | Proteus mirabilis strain ATCC 7002 |
| AORN01000003.1 | Proteus mirabilis PR03 |
| KB206033.1 | Proteus mirabilis C05028 |
| JH815534.1 | Proteus mirabilis WGLW4 |
| JH815507.1 | Proteus mirabilis WGLW6 |
| GG668581.1 | Proteus mirabilis ATCC 29906 |
| AM942759.1 | Proteus mirabilis strain HI4320 |
| FOPN01000020.1 | Proteus mirabilis strain NLAE-zl-G534 |
| MCOR01000055.1 | Proteus mirabilis strain 1091 |
| LGTA01000022.1 | Proteus mirabilis strain Pr2921 |
| LQQZ01000009.1 | Proteus mirabilis strain M16 |
| JSUP01000105.1 | Proteus mirabilis strain PM593 |
| JVHI01000059.1 | Proteus mirabilis strain 430_PMIR |
| JVHK01000013.1 | Proteus mirabilis strain 429_PMIR |
| JUXK01000061.1 | Proteus mirabilis strain 68_PMIR |
| JVFU01000032.1 | Proteus mirabilis strain 47_PMIR |
| JVOK01000170.1 | Proteus mirabilis strain 25_PMIR |
| JVEH01000082.1 | Proteus mirabilis strain 51_PMIR |
| JVWE01000043.1 | Proteus mirabilis strain 127_PMIR |
| JWBG01000095.1 | Proteus mirabilis strain 1150_PMIR |
| JWBY01000040.1 | Proteus mirabilis strain 1134_PMIR |
| JVJQ01000135.1 | Proteus mirabilis strain 373_PMIR |
| JSUO01000150.1 | Proteus mirabilis strain PM655 |
| CP004022.1 | Proteus mirabilis BB2000 |
| LTBK01000025.1 | Proteus mirabilis strain Wood |
| LANL01000032.1 | Proteus mirabilis strain 25933 |
| LDIU01001573.1 | Proteus mirabilis strain SAS71 |
| LUFT01000033.1 | Proteus mirabilis strain SCDR1 |
| LNHT01000086.1 | Proteus mirabilis strain 50664164 |
| JVUH01001687.1 | Proteus mirabilis strain 1310_PMIR |
| KN150746.1 | Proteus vulgaris strain ATCC 49132 |
| LXEV01000011.1 | Proteus hauseri ATCC 700826 |
| CVRZ01000021.1 | Proteus vulgaris genome assembly PRJEB9439 |
| AWXP01000023.1 | Proteus hauseri ZMd44 |
| CVRY01000006.1 | Proteus vulgaris genome assembly PRJEB9438 |
| GG661996.1 | Proteus penneri ATCC 35198 |
