# Supplementary material for: Genetic diversity of the O antigens of Proteus species and the development of a suspension array for molecular serotyping
Source: PLoS One. 2017 Aug 17;12(8):e0183267. doi: 10.1371/journal.pone.0183267 (PMC5560731; doi:10.1371/journal.pone.0183267)
Supplement: S7 Table — (DOC) [file pone.0183267.s007.doc]

**S7 Table. The GTs and HGs.**

| **Homology Groups** | **Strains and GT Names** | **Fuctions** |
| --- | --- | --- |
| HG01 | O3ab-P.m-G2292_gt5 O5-P.m-G2609_gt5 O13-P.m-G2616_gt3 O14ab-P.m-G2617_gt4 O16-P.m-G3924_gt3 O17-P.v-G2619_gt5 O18-P.m--G2621_gt4 O24-P.m-G2627_gt4 O29a-P.m-G2630_gt4 O30-P.m-G2631_gt4 O40-P.m-G2639_gt3 O45-P.v-G2642_gt6 O50-P.m-G2647_gt4 O52-P.v-G4071_gt3 O53-P.v-G2648_gt2 O56-P.g4-G2652_gt4 |  |
| HG02 | O6-P.m-G2610_gt3  O8-P.v-G2612_gt3  O12-P.v-G2615_gt4 O19a-P.v-G2622_gt3 O42-P.v-G2300_gt3 | WbuB *E.coli*  ADI43268.1; Id:62-65%  putative L-fucosamine transferase  α-L-FucNAc-(1→3)- D-GlcNAc |
| HG03 | O10-P.m-G2294_gt1 O23ac-P.v-G2297_gt1 O26-P.m-G2629_gt1 O28-P.m-G2299_gt1 O41-P.m-G2640_gt1 O51-P.m-G2646_gt1 O71-P.p-G2669_gt1 | WenA *P.vulgaris*  ADL32305.1; Id:89-100% |
| HG04 | O10-P.m-G2294_gt2 O23ac-P.v-G2297_gt2  O26-P.m-G2629_gt2  O28-P.m-G2299_gt2  O41-P.m-G2640_gt2  O51-P.m-G2646_gt2 O71-P.p-G2669_gt2 | WenB *P.vulgaris*  ADL32306.1; Id:75-100% |
| HG05 | O5-P.m-G2609_gt4  O10-P.m-G2294_gt6 O23ac-P.v-G2297_gt3  O27-P.m-G2298_gt6 O45-P.v-G2642_gt5  O74-P.m-G2674_gt4 | WemV *P.mirabilis*  ADL32326.1; Id:96-100%  α-D-GalA-(1→3)- D-GlcNAc |
| HG06 | O34-P.v-G2635_gt3 O41-P.m-G2640_gt5 O54ab-P.m-G2649_gt2 O57-P.m-G2653_gt4  O73ab-P.p-G2672_gt2 | AmsE *P.mirabilis*  WP_049256413.1; Id:76-100%  amylovoran biosynthesis protein AmsE  β-D-Gal-(1→3)- D-GalNAc |
| HG07 | O6-P.m-G2610_gt1  O11-P.m-G2614_gt1 O20-P.m-G2624_gt1 O57-P.m-G2653_gt1 |  |
| HG08 | O17-P.v-G2619_gt4 O20-P.m-G2624_gt4 O37ab-P.v-G2636_gt4 O69-P.p-G2667_gt3 | Acinetobacter baumannii  WP_023060370.1; Id:55-57%  N-acetylgalactosaminyl-diphosphoundecaprenol glucuronosyltransferase |
| HG09 | O17-P.v-G2619_gt1 O37ab-P.v-G2636_gt1  O45-P.v-G2642_gt1 |  |
| HG10 | O29a-P.m-G2630_gt3 O44-P.v-G2641_gt4 O56-P.g4-G2652_gt3 | WemE *P.mirabilis*  ADL32281.1; Id:73-100% |
| HG11 | O9-P.m-G2613_gt1  O36-P.m-G3926_gt1 | β-D-Gal-(1→3)- D-GlcNAc |
| HG12 | O12-P.v-G2615_gt3 O19a-P.v-G2622_gt2 | an N-acetyl-L-fucosamine (L-FucNAc) transferase  α-L-FucNAc-(1→3)- D-GlcNAc |
| HG13 | O14-P.m-G2617_gt1 O41-P.m-G2640_gt3 | β-D-Gal-(1→3)- D-GalNAc |
| HG14 | O14-P.m-G2617_gt3 O18-P.m--G2621_gt3 |  |
| HG15 | O16-P.m-G3924_gt2 O48-P.m-G2644_gt2 | PglA *Citrobacter europaeus* SBW26876.1; Id:55% Alpha-1,3-N-acetylgalactosamine transferaseα-D-GalNAc-(1→3)- D-GlcNAc |
| HG16 | O27-P.m-G2298_gt5 O74-P.m-G2674_gt3 | α-D-GalA6(L-Ala)-(1→3)- D-GlcNAc |
| HG17 | O30-P.m-G2631_gt1  O60-P.myx-G2656_gt2 | α-D-GalNAc-(1→6)- D-GlcNAc  or  β-D-GlcNAc-(1→3)- D-GlcNAc  or  β-D-GlcNAc-(1→4)- D-GlcA |
| HG18 | O30-P.m-G2631_gt2  O60-P.myx-G2656_gt3 |
| HG19 | O30-P.m-G2631_gt3  O60-P.myx-G2656_gt4 |
