# Supplementary material for: Genetic diversity of the O antigens of Proteus species and the development of a suspension array for molecular serotyping
Source: PLoS One. 2017 Aug 17;12(8):e0183267. doi: 10.1371/journal.pone.0183267 (PMC5560731; doi:10.1371/journal.pone.0183267)
Supplement: S8 Table — The amino acid sequence identities were obtained in comparison with the E. coli K12 Wzz. (DOC) [file pone.0183267.s008.doc]

**S8 Table. The Wzz in the 68 *Proteus* genomes. The amino acid sequence identities were obtained in comparison with the *E. coli* K12 Wzz.**

| **Genome ID** | **Identities** |
| --- | --- |
| JVUH01003963.1 | 100 |
| FOPN01000020.1 | 99.72 |
| FNAA01000018.1 | 99.72 |
| CP020052.1 | 99.72 |
| MCOR01000055.1 | 99.72 |
| MBTY01000036.1 | 99.72 |
| LWUM01000125.1 | 99.72 |
| LWUL01000070.1 | 99.72 |
| LGTA01000022.1 | 99.72 |
| LTBK01000025.1 | 99.72 |
| CP015347.1 | 99.72 |
| LWDB01000010.1 | 99.72 |
| LQQZ01000009.1 | 99.72 |
| LQNO01000021.1 | 99.72 |
| LQNN01000019.1 | 99.72 |
| KV388090.1 | 99.72 |
| KQ960990.1 | 99.72 |
| JSUO01000150.1 | 99.72 |
| JSUP01000105.1 | 99.72 |
| LGAY01000041.1 | 99.72 |
| CP012675.1 | 99.72 |
| CP012674.1 | 99.72 |
| JUYT01000059.1 | 99.72 |
| JVHI01000059.1 | 99.72 |
| JVHK01000013.1 | 99.72 |
| JVHX01000049.1 | 99.72 |
| JVJQ01000135.1 | 99.72 |
| JUXK01000061.1 | 99.72 |
| JVFU01000032.1 | 99.72 |
| JVMQ01000065.1 | 99.72 |
| JVOK01000170.1 | 99.72 |
| JVEH01000082.1 | 99.72 |
| JVKD01000015.1 | 99.72 |
| JVPB01000062.1 | 99.72 |
| JVTJ01000130.1 | 99.72 |
| JVUE01000042.1 | 99.72 |
| JWAP01000093.1 | 99.72 |
| JVTO01000142.1 | 99.72 |
| JVVD01000056.1 | 99.72 |
| JVWE01000043.1 | 99.72 |
| JWBG01000095.1 | 99.72 |
| JWBY01000040.1 | 99.72 |
| JWCS01000112.1 | 99.72 |
| LANL01000032.1 | 99.72 |
| JTBP01000001.1 | 99.72 |
| JTBB01000001.1 | 99.72 |
| JTBA01000001.1 | 99.72 |
| JTAW01000004.1 | 99.72 |
| JSCB01000061.1 | 99.72 |
| KN150749.1 | 99.72 |
| CP004022.1 | 99.72 |
| AORN01000003.1 | 99.72 |
| KB206033.1 | 99.72 |
| JH815534.1 | 99.72 |
| JH815507.1 | 99.72 |
| AM942759.1 | 99.72 |
| JUXR01000308.1 | 99.43 |
| JVXV01000113.1 | 99.43 |
| GG668581.1 | 99.43 |
| LNHT01000086.1 | 99.15 |
| LUFT01000033.1 | 99.15 |
| CVRZ01000021.1 | 90.93 |
| KN150746.1 | 90.65 |
| AWXP01000023.1 | 89.52 |
| LXEV01000011.1 | 89.49 |
| CVRY01000006.1 | 88.92 |
| GG661996.1 | 88.67 |
| LDIU01001574.1 | 86.16 |
